# Levetiracetam Induced Psychosis – A Case Study

**DOI:** 10.1192/bjo.2025.10761

**Published:** 2025-06-20

**Authors:** Saikat Roy, Akshay Dixit

**Affiliations:** 1 Cassel Hospital, London, United Kingdom; 2 Collingham Child and Family Centre, London, United Kingdom

## Abstract

**Aims:** Levetiracetam is a broad spectrum antiepileptic used in a variety of seizure disorders in both adults and children. Although a popular antiseizure medication, levetiracetam’s association with new onset behavioural disturbance such as agitation, hostility, psychosis and mood symptoms has been widely reported in scientific literature. Seizure disorders themselves can present with psychiatric manifestations. We are reporting a case of an adolescent male where the interphase of physical and mental health came into play.

**Methods:** A 13-year-old male presented to A&E brought by his family following a referral from the epilepsy clinic due to two weeks history of bizarre behaviours including abnormal gait, tapping on the shoulders of his family members, talking to himself and generally being more irritable. From history, we noted he had been diagnosed with epilepsy (unspecified) for two years and recently his seizure activities increased in frequency, which prompted his neurologist to increase his antiseizure medication (levetiracetam from 1250 mg twice a day to 1500 mg twice a day) two weeks prior to his presentation, which coincided with the onset of his symptoms.

He reported experiencing intrusive and unpleasant thoughts about the safety of his family, experiencing multiple times of the day and to reduce the anxiety he was tapping on their shoulder, and checking the locks of the door and windows of the house, the thoughts and rituals corresponded to obsession and compulsion. He also reported thought broadcasting – people are able to know what he was thinking, and abnormal perception of hearing his own thoughts spoken aloud – appeared to be Gedankenlautwerden.

In the emergency department he underwent extensive blood (including auto-antibodies associated with first episode of psychosis) and radiological investigations to rule out acute neurological causes. The investigations did not yield any positive results, his levetiracetam level was also within therapeutic range.

The description of his seizures indicated that he experiences gustatory and olfactory auras with focal to generalised seizures followed by postictal transient paresis of the left arm, which has been consistent over the course of the two years he had the seizure.

**Results:** Diagnostic formulation was the acute onset obsessive-compulsive and psychotic symptoms are likely the direct result of the increase in the dose of levetiracetam which had a temporal relationship, differentials included psychiatric symptoms associated with epilepsy: we queried temporal lobe, and a functional psychotic illness. We advised for a medication review, and his levetiracetam was reduced to a prior dose and lamotrigine was added on by the epilepsy clinic.

**Conclusion:** Two months after the presentation we received a letter from his paediatrician mentioning his psychiatric symptoms have improved and the add-on medication managed to control his seizure.

